# Evaluation of Adhesive Seams of High-Density Polyethylene Geomembrane Subjected to Wetting and Freeze-Thaw Cycles

**DOI:** 10.3390/ma18102368

**Published:** 2025-05-20

**Authors:** Xianlei Zhang, Jialong Zhai, Yuan Tang, Yunyun Wu

**Affiliations:** 1 School of Water Conservancy, North China University of Water Resources and Electric Power, Zhengzhou 450046, China; zhangxianlei@ncwu.edu.cn (X.Z.);; 2 Chengdu Engineering Corporation Limited, Chengdu 610072, China; 3 College of Water Conservancy and Hydropower Engineering, Hohai University, Nanjing 210024, China

**Keywords:** geosynthetics, HDPE, bonding, seam strength, seam efficiency

## Abstract

The seaming of geomembranes (GMBs) is a critical aspect of their successful functioning as barriers to liquid, with bonding and welding being the commonly employed methods. Due to the limitations of conventional welding methods at the connection points between the geomembrane and the structure, extrusion welding often results in damage at the seams. The bonding method, which has lower requirements for construction conditions, has emerged as a currently viable alternative seaming technique. Bonding techniques are widely applied in small reservoirs and embankments. This study investigates the performance of high-density polyethylene (HDPE) GMB seams bonded using asphalt-based adhesive (ABA) and non-asphalt-based adhesive (NABA). Seam tensile tests were conducted under wetting and freeze-thaw cycles (FTCs) conditions to evaluate the mechanical properties of the seamed GMBs. The results indicated that the seam strength of specimens bonded with ABA increased as wetting time and FTCs increased (with a maximum increase of 113.8%). In contrast, specimens bonded with NABA exhibited decreased seam strength under similar conditions (with a maximum decrease of 93.4%). Both types of specimens exhibited enhanced seam strength with increasing seam width. Due to wetting and FTCs, the seam efficiency of NABA-bonded specimens decreased, while that of ABA-bonded specimens showed slight improvement. However, the improved seam efficiency remained below 1.2%, an extremely small value. The axial tensile strength of bonded specimens was significantly lower than that of seamless specimens, failing to fulfill long-term safety operation requirements. Therefore, bonding method should be used cautiously at non-critical structural components where the welding is impractical but repair and replacement are relatively simple. The findings provide insight for GMB installers and design engineers in order to improve the performance of HDPE GMB seams.

## 1. Introduction

High-density polyethylene (HDPE) geomembranes (GMBs) have been extensively utilized as primary anti-seepage materials in water conservancy, environmental and geotechnical engineering for their advantages of excellent anti-seepage performance, ease of construction, and low engineering costs [1,2,3,4,5,6,7]. However, the width of a single roll, generally 6 m to 10 m, fails to meet the anti-seepage requirements for reservoir or dam surfaces. Consequently, field seaming is the predominant technique employed to address this limitation. GMB seams represent critical weak points and potentials source of leakage, and many problems (e.g., stress cracking) encountered originate at these seam locations [8]. Rollin et al. [9] reported that 55% of damage recorded in exposed GMB liners occurred at seams. Furthermore, Gassner and Fairhead [10] noted that 32% of recorded defects were attributed to defective seams. Fan and Rowe [11] reported that the elastomeric bituminous geomembrane seams are highly susceptible to creep rupture under sustained tensile loads. Furthermore, they found that both the time to rupture and strain at rupture for acceptable welds are both exponentially correlated with the sustained load. Therefore, it is imperative to ensure the highest quality in seam creation.

The seaming of GMB rolls and panels is a critical aspect of their successful functioning as a barrier to liquid, with bonding and welding being the commonly employed methods [12]. Giroud [13] noted that the failure of a GMB next to a seam resulted from stress concentration, typically caused by thickness reductions. Giroud et al. [14] provided equations to calculate the strain concentration with parameters such as GMB thickness, modulus, seam type, seam thickness, and seam width. Kavazanjian et al. [15] conducted laboratory testing using digital image correlation to evaluate strain concentrations adjacent to HDPE GMB seams. Regarding research on welding method, Stark et al. [16] concluded that factory-welded seams exhibited higher seam peel and shear strengths at yield than field-welded thermal seams. Rowe and Shoaib [17] reported on the long-term performance of dual wedge welds in an HDPE GMB when exposed to synthetic municipal solid waste leachate. Shoaib and Rowe [18] investigated the durability and long-term strength of seams in HDPE geomembranes. Zhang et al. [19] examined the effect of dual-wedge welding parameters on the physical, mechanical, and chemical properties of HDPE GMB seams. Rowe and Shoaib [20], examined the depletion of antioxidants and mechanical properties for one weld immersed in a synthetic leachate solution. Francey and Rowe [21] evaluated the seam squeeze-out antioxidant loss and thickness reduction for three HDPE GMBs seamed using a variety of welding parameters and two different wedge welders. Peggs [22] assessed the implications of seam squeeze-out beads regarding craze formation and seam stress crack resistance and concluded that a low Std-OIT squeeze-out bead may induce stress cracking within the heat affected zones. Francey and Rowe [23] investigated the stress crack resistance of HDPE GMB fusion seams, including unnotched and notched seams, across a range of welding parameters, and identified deleterious squeeze-out geometries. Francey and Rowe [24] examined the effects of seam thickness reduction and overlap width on seam strength of bituminous geomembrane seams. In terms of research on bonding method, Thomas et al. [25] studied thermally bonded PVC geomembrane seams, and developed a relationship between seam peel strength and burst pressure, enabling non-destructive quality assurance/quality control operations in the field. Stark et al. [26] refined the relationship between the required air channel pressure and the sheet temperature. Stark and Pazmino [27] conducted research on high-temperature air channel testing of thermally bonded PVC geomembrane seams. Owing to the excessively high welding temperature, the application of welding methods has led to mechanical damage of GMBs at the seams and their surrounding areas, thereby forming a weak link in the impermeable system. This issue is particularly pronounced at the connection points between the GMB and the structure. Whereas bonding method generally does not cause damage to the materials during the construction process. However, the physical and mechanical properties of the seams at the bonding areas are the key points for quality control. Previous studies have primarily focused on the welded seams of HDPE GMBs, however, literature regarding bonded seams is predominantly related to PVC GMBs. Consequently, there remains a paucity of research on bonded seams of HDPE GMBs.

The objective of this paper is to evaluate the mechanical properties of adhesive seams in HDPE GMBs subjected to wetting and freeze-thaw cycles (FTCs). The objectives are structured as follows: (1) to select an appropriate type of seamed specimen and tensile rate for seam tensile tests; (2) to investigate the effect of wetting time and FTCs on the seam strength of bonded specimens; (3) to assess the seam efficiency of bonded specimens under wetting and FTCs conditions; (4) to compare the results between bonded specimens and seamless specimens, and analyze the practical feasibility of bonding techniques in engineering applications.

## 2. Materials and Methods

### 2.1. Test Materials

Commercially available HDPE GMBs (Shandong Jiantong New Materials Technology Group Co., Ltd., Dezhou, China) were employed for seam tensile tests. GMB specimens were bonded with two commonly used special adhesives: asphalt-based adhesive (ABA), primarily used in embankment and reservoir applications, and non-asphalt-based adhesive (NABA), mainly applied in civil engineering projects. The basic parameters of test materials measured according to ASTM D5261-10, ASTM D5199-12 and ASTM D4885-01 [28,29,30] are summarized in Table 1.

### 2.2. Test Methods

#### 2.2.1. Standard Method

**Figure 1:**
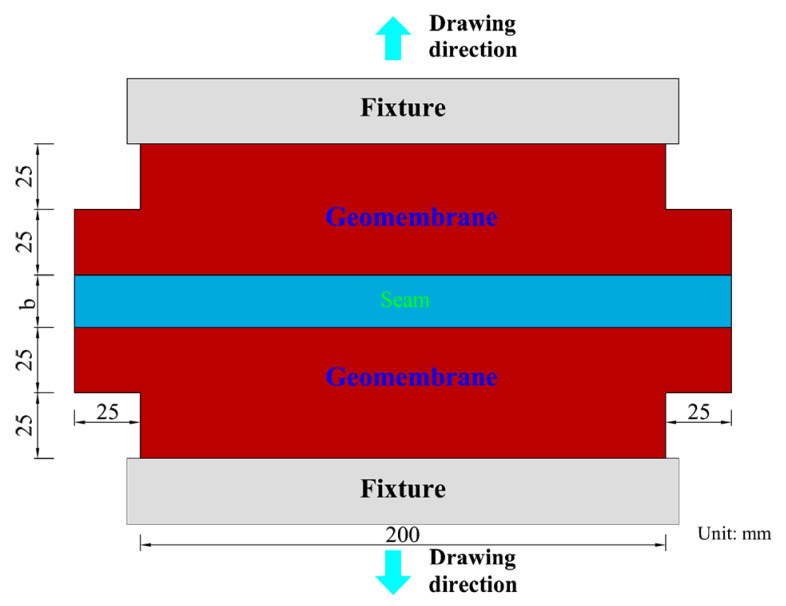
Specimen by standard method: b is the seam width.

The seamed specimens were prepared in accordance with GB/T 13760-2009 and SL 235-2012 [31,32], as illustrated in Figure 1. In accordance with ASTM D4885-01 [30], Wide strip tensile tests were conducted using a tensile testing machine for geosynthetics (TTMG) (CMT-5000, Nss (Shenzhen) Laboratory Equipment Co., Ltd., Shenzhen, China) at a tensile rate of 2 mm/min and at a temperature of 20 ± 2 °C. The typical parameters of TTMG are as follows: a maximum load of 30 kN, a maximum range of 2.1 m, and displacement ranging from 0.2% to 100% of the maximum stroke with an error of 0.5%.

**Figure 2:**
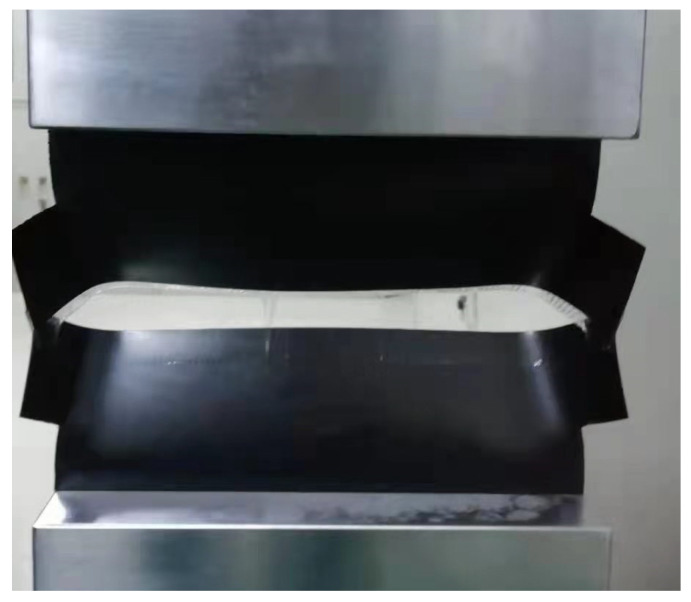
Yield failure form by standard method.

Figure 2 presents the morphology of the bonded specimen at failure according to standard method. As depicted, within the fixture width, the upper and lower GMB in the bonding area had completely separated. In contrast, beyond the fixture width, the GMBs had not completely separated, and exhibited transverse tearing and axial tensile failure due to the axial tensile force within the fixture width. During the testing process, it was observed that the initial separation of the adhesive surface occurred in the middle of the fixture width, and then expanded progressively toward both sides until complete separation was achieved. This phenomenon indicates that prior to the failure of the outward-extending parts, the middle core analysis area in the standard specimen had already sustained damage. The region beyond the bonded area influences not only the axial tensile morphology but also the maximum axial tensile force within the test width. Therefore, the results cannot accurately reflect the mechanical properties of the bonding surface. Thus, to accurately evaluate the performance of seamed GMBs, it is necessary to modify the specimen design based on the specification.

#### 2.2.2. Modified Method

**Figure 3:**
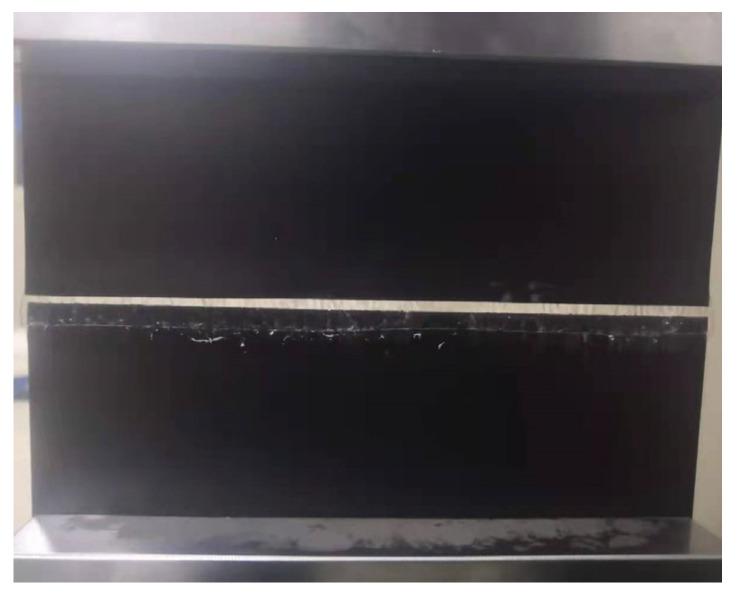
Yield failure form by modified method.

In view of the limitations of the standard method, this study improved the specimen design based on the standard specimen. The modified specimens eliminated the outwards-extending parts on both sides of the bonding area as stipulated in the standard. After bonding, the specimens were rectangular, with a width of 200 mm and a length of 100 mm excluding the bonding section. During the axial tensile test, the displacement of the modified specimen changed uniformly in the bonded part. When failure in the bonding part occurred, the bonding surface detached simultaneously, as shown in Figure 3. Notably, there was no tearing phenomenon originating from the central region and propagating to both sides. In the modified specimens, the outward-extending parts have been removed, allowing for overall failure to occur at the seam locations. Consequently, the modified specimen can accurately reflect the mechanical properties of the bonding interface.

**Figure 4:**
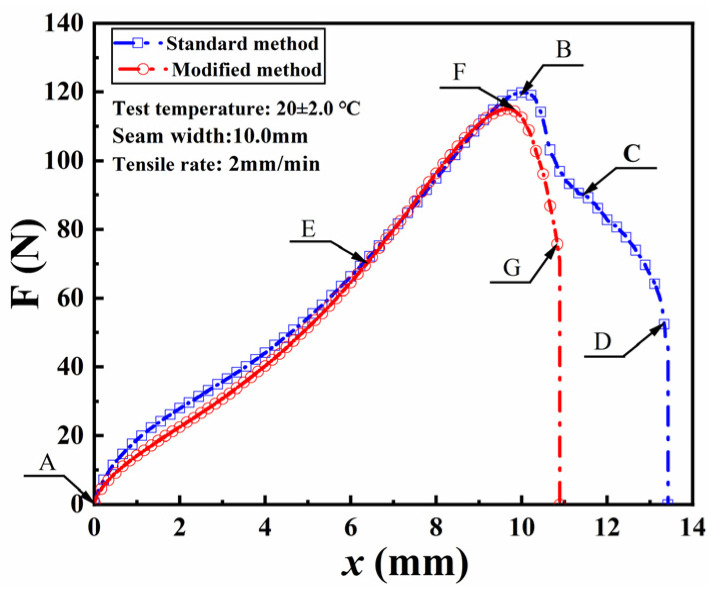
Axial force-displacement curves of standard and modified method.

Figure 4 displays the axial tension-displacement curves of seamed specimens bonded with NABA, denoted as A-E-B-C-D for the standard specimen and A-E-F-G for the modified specimen. Initially, the axial tension exhibited a linear relationship with displacement (lines AF and AB), and then the curves diverged significantly. Within the AE interval, the axial tension of standard specimen was slightly higher than that of modified specimen at the same displacement. The initial difference in axial tension can be attributed primarily to the greater shear force induced at the adhesive-GMB interface due to the larger bonded area in the standard specimen compared to the modified specimen. However, as tensile displacement increased, the influence of shear force diminished, resulting in a strong correlation between curves EF and EB. Notably, the maximum axial tension for the modified specimen occurred earlier than that for the standard specimen, suggesting that the extended sections at the seams of the standard specimen influenced the test results.

After reaching the maximum axial tension, the standard specimen showed a gradual decline in axial tension (i.e., lines BC and CD), whereas the modified specimen exhibited a sharp decrease (i.e., line FG) until failure occurred at the seams. However, both specimens experienced detachment at the interface between the adhesive and the GMB at failure. After the detachment in the width range, the standard specimen completely detached at point C due to the extended sections at the seams, resulting in deviations from the behavior of the modified specimen. Therefore, the interface shear force at the extended sections was primarily responsible for the slower reduction rate of axial tension in the standard specimen.

In conclusion, the extended sections at the seams of the standard specimen affected the accuracy of the test results, leading to higher values for both the axial tension and the tensile displacement corresponding to its maximum. In contrast, the modified specimens demonstrated uniform tensile failure at the seams, providing more representative and reliable test outcomes. Therefore, this study adopted the modified specimens for further analysis.

### 2.3. Selection of Tensile Rate and Seam Width

Various tensile rates for seam tensile tests were recommended in existing test methods, as described in Table 2. However, these rates were primarily intended for material quality assessment and offer limited practical guidance. During the construction and operation of GMBs, tensile deformations at higher rates were unlikely to occur unless there was non-compliance with construction standards or exceptional operational circumstances. Zhao et al. [33] reported that the tensile strain rates significantly influence the tensile load, with higher strain rates resulting in increased tensile loads and more linear load-strain curves of polymer-blend geocell sheets. Considering the differential settlement observed in existing water conservancy projects, three tensile rates—2.0 mm/min, 5.0 mm/min, and 10.0 mm/min—were selected to evaluate their impact on axial tensile mechanical properties. Furthermore, two adhesive materials, ABA and NABA, were utilized in the study.

The mechanical properties at the seams under axial tensile loading were primarily characterized by the shear stress on the interface between the adhesive and the GMB. Therefore, the seam width was a critical factor influencing these properties. In the experimental setup, three different ratios of seam width to gauge length were investigated: 10%, 20%, and 30%, corresponding to seam widths of 10 mm, 20 mm, and 30 mm, respectively.

**Figure 5:**
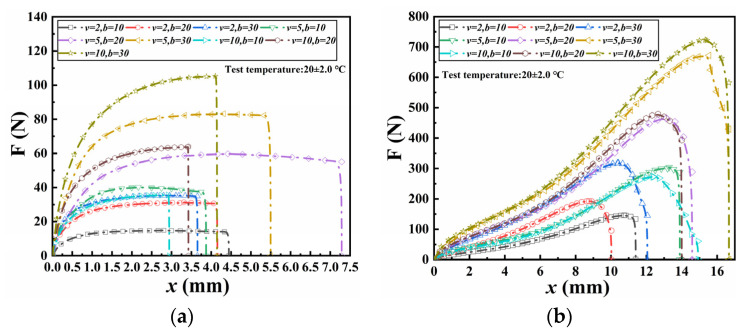
Relationship between axial force and displacement at different tensile rates: (**a**) ABA; (**b**) NABA.

The tensile tests were initially performed to determine a suitable tensile rate. Figure 5 presents the relationship between axial tension and displacement at different tensile rates for specimens bonded with ABA and NABA, respectively. For ABA-bonded specimens, axial tension-displacement curves initially demonstrated rapid growth, subsequently reaching a peak tension before exhibiting a sharp decline. For specimens with identical seam widths, both axial tension and peak tension increased with increasing tensile rates. In contrast, for NABA-bonded specimens, axial tension-displacement curves exhibited an initial gradual growth followed by a fast decline until failure occurred. For specimens with the same seam width, higher tensile rates resulted in increased axial tension, peak tension values. At the same seam width and tensile rate, the peak tension of NABA-bonded specimens was significantly higher than that of ABA-bonded specimens.

Consequently, the tensile rate had a significant influence on the results, leading to higher peak tensions at elevated tensile rates. In engineering applications, the tensile rates of GMBs were influenced by the settlement difference and settlement time of bearing layers. A greater settlement difference occurring over a shorter period resulted in a higher tensile rate for the GMBs. Significant settlement differences were observed at the peripheral joints, with slightly larger values at the initial stage of reservoir impoundment and relatively smaller values during operation. However, the settlement rates in both periods were slower than the tensile rates used in the tests. A statistical analysis was conducted on the initial impoundment settlement of high concrete-faced rockfill dams in China. The results indicated that, except for the Tianshengqiao Level 1 dam, the maximum settlement for most dams did not exceed 36.1 cm. The minimum storage time varied from several days to dozens of days. Assuming an initial storage time of 2 h, the average settlement rate was calculated to be 0.125 mm/min, lower than the minimum tensile rate used in the tests. In practice, a longer initial storage time resulted in a significantly slower settlement rate. However, due to limitations in testing conditions, extremely low tensile rates were impractical. To provide valuable reference for engineering applications, a tensile rate of 2 mm/min was recommended as appropriate.

The experimental data demonstrated that, at a constant tensile rate, the peak tension increased proportionally with each increment in seam width. The interface characteristics between the adhesive and the GMB were influenced by both the seam width and the gauge distance between fixtures. An increase in the ratio of seam width to specimen gauge length resulted in higher maximum tension but also incurred greater costs. Therefore, the seam width should be determined reasonably according to engineering practice and test results. At the same tensile rate and seam width, specimens bonded with NABA exhibited significantly higher maximum tension compared to those bonded with ABA, indicating superior mechanical properties for NABA-bonded specimens in a dry environment at room temperature.

### 2.4. Seam Tensile Test Under Wetting Conditions

Since the seamed GMBs by adhesives are subjected to prolonged immersion in water during operation, wetting may result in a reduction in seam strength, thereby affecting the safe operation of the project. In this paper, wetted specimens were prepared to simulate the immersion environment, and tensile tests were performed in accordance with ASTM D4885-01 to investigate the effect of wetting on the seam strength.

According to different seam widths, the width of wetting specimens was set to 110, 120, and 130 mm respectively. Two types of adhesives, namely ABA and NABA, were selected. Fifteen specimens with the same seam width were prepared for each type of adhesive. Considering the maximum potential load during the actual engineering operation, the seamed area of each specimen was subjected to static compression by a 30 kg weight for 24 h. Subsequently, the specimens were immersed in a distilled water tank maintained at a constant temperature of 20 ± 2 °C for 10, 20, and 30 d. Five specimens with the same adhesive and seam width were taken at each immersion time for the seam tensile test at a tensile rate of 2 mm/min.

### 2.5. Seam Tensile Test Under FTCs Conditions

GMBs were employed as anti-seepage materials on the upstream face of the reservoir or water storage structures. During operation, FTCs may induce changes in the freeze-thaw resistance of specimens in the bonded areas, potentially shortening their service life. To reveal the impact of FTCs on seam strength and evaluate seam quality, freeze-thaw cycling tests were conducted on specimens bonded with two types of adhesives as per ASTM D4885-01.

The test equipment selected was a freeze-thaw tester (model HDK-9, manufactured by a laboratory in Cangzhou, Cangzhou, China) in hydraulic structure laboratory. Its main parameters are as follows: the freeze-thaw cycle period ranges from 2.5 to 5.5 h, the low-temperature control range is −23 to −18 °C, the high-temperature control range is 18 to 23 °C, and the heating power is 3 kW. The refrigeration system employs a fully enclosed 4HP compressor.

The form and number of bonded specimens for freeze-thaw tests were identical to those used in wetting tests, and five seamless specimens were selected for each FTC. The low temperature was controlled at −18 ± 2 °C, while the high temperature was set at 20 ± 2 °C. The heating time was 1.0 h, the cooling time was 1.5 h, and one complete FTC lasted 5.0 h. The specimens underwent 100, 150, and 200 FTCs, respectively. After completing the specified number of FTCs, the specimens were taken out, and the water on their surface was wiped clean. Subsequently, five specimens with the same number of FTCs, type of adhesive, and seam width were selected for seam tensile tests.

### 2.6. Analysis Method in Specification

The joint/seam strength and joint/seam efficiency are typically employed to evaluate the mechanical properties of bonded specimens [32,34], and the joint/seam strength is expressed as follows:

(1) $$T_{Smax}=\frac{F}{B}$$

where $T_{Smax}$ is the joint/seam strength, F is the maximum failure load, and B is the width of the specimen.

The joint/seam efficiency is defined as:

(2) $${\&}_{S}=\frac{100\times T_{Smax}}{T_{max}}$$

(3) $$T_{max}=\frac{F}{B_{0}}$$

where ${\&}_{S}$ is the joint/seam efficiency, $T_{max}$ is the average tensile strength of the seamless specimen, and $B_{0}$ is the width of the seamless specimen.

## 3. Results

### 3.1. Effect of Wetting Time and Seam Width on Seam Strength

**Figure 6:**
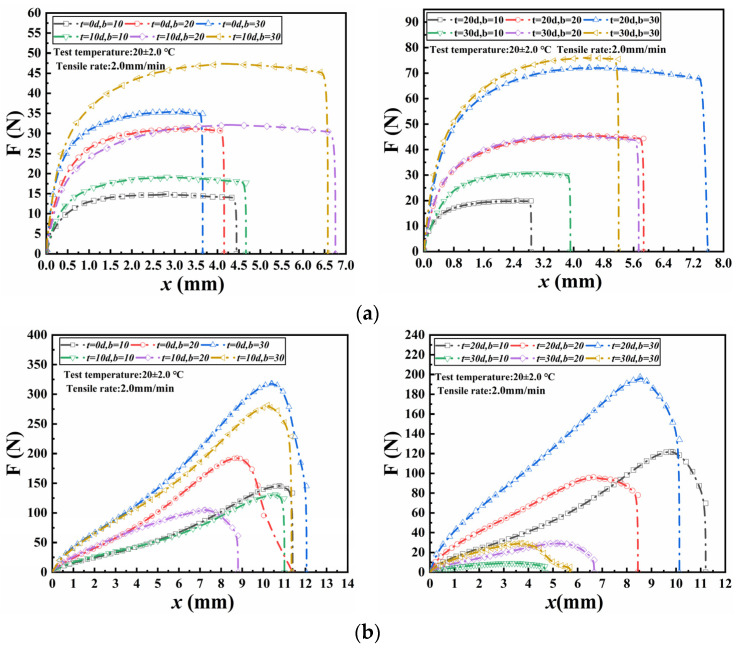
Relationship between axial force and displacement of wetted specimens: (**a**) ABA; (**b**) NABA.

Figure 6 depicts the relationship between axial tension and tensile displacement for wetted specimens during the seam tensile test. As observed, the curves of axial tension versus displacement for wetted specimens followed similar patterns to those of non-wetted specimens. For seamed specimens bonded with ABA, the axial tension increased rapidly at the initial stage and stabilized for a long period. For seamed specimens bonded with NABA, the axial tension rose sharply until it reached the maximum, after which it declined. Under identical conditions, i.e., the same wetting time and seam width, both the peak axial tension and the corresponding tensile displacement of NABA-bonded specimens were larger than those of ABA-bonded specimens. Under the same wetting time, the peak axial tension of wetted specimens increased proportionally with each increment in seam width. For a given seam width, as the wetting time increased, the peak axial tension also increased.

Table 3 lists the seam strength calculated using Equation (1) and the differences in seam strength resulting from varying wetting times. For specimens bonded with ABA, the seam strength increased gradually as wetting time increased, eventually reaching a stable level. For example, at 30 days of wetting, the seam strength exhibited respective increases of 86.8%, 93.5%, and 113.8% for seam widths of 10 mm, 20 mm, and 30 mm. This increase was also positively correlated with seam width. In contrast, for specimens bonded with NABA, the seam strength exhibited a gradual decrease with the increasing wetting time. Likewise, at 30 days of wetting, the seam strength decreased by 93.4%, 84.8% and 90.8%, respectively when the seam width was 10 mm, 20 mm and 30 mm. Within the same period of wetting time difference, the reduction in seam strength showed an increasing trend with larger seam widths experiencing a steeper decline gradient. Initially, the seam strengths of NABA-bonded specimens were significantly higher than those of ABA-bonded specimens. Due to the decrease in seam strength of NABA-bonded specimens while the increase in seam strength of ABA-bonded specimens with increasing wetting time, the former seam strength became lower than the latter under the same width after 30 days of wetting.

### 3.2. Effect of FTCs and Seam Width on Seam Strength

**Figure 7:**
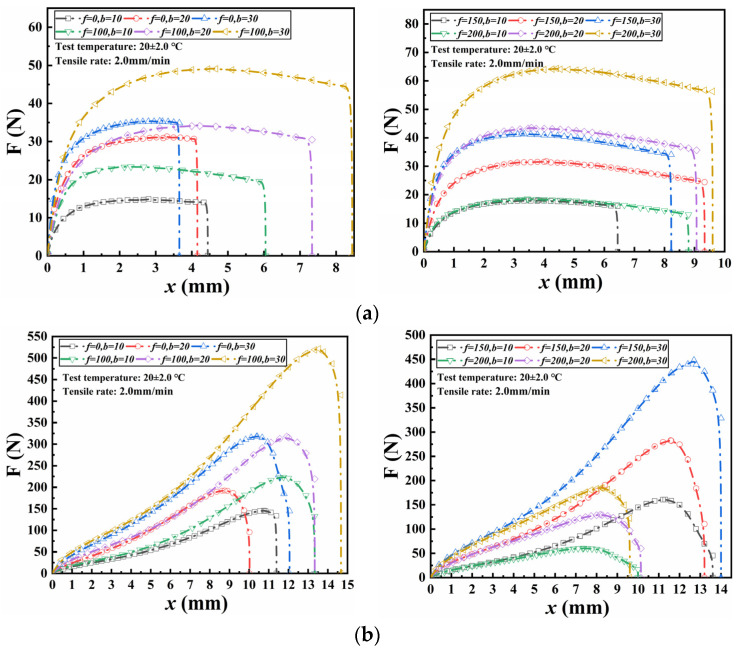
Relationship between axial force and displacement subjected to FTCs: (**a**) ABA; (**b**) NABA.

Figure 7 presents the relationship between axial tension and tensile displacement for seamed specimens subjected to FTCs during seam tensile tests. The curves of axial tension and displacement for specimens prior to FTC exposure exhibited a close correlation with those subjected to FTCs. For seamed specimens bonded with ABA, the axial tension initially increased rapidly before stabilizing over an extended period. In contrast, for seamed specimens bonded with NABA, the axial tension gradually increased until approaching a peak, after which it sharply declined. Under identical conditions, including the same FTCs and seam width, both the peak axial tension and corresponding tensile displacement were greater for specimens bonded with NABA compared to those bonded with ABA. Additionally, under the same FTC conditions, the peak axial tension increased proportional to every increase of seam width. Furthermore, for a given seam width, the peak axial tension also increased as the number of FTCs increased.

The seam strength calculated using Equation (1) and the difference in seam strength produced by different FTCs are detailed in Table 4. As the number of FTCs increased, the seam strength of ABA-bonded specimens initially increased, followed by a decrease, and then increased again. In contrast, the seam strength of NABA-bonded specimens increased and then decreased with the increasing number of FTCs. For both specimens, it consistently increased with the increasing seam width.

Subjected to FTCs, specimens bonded with NABA exhibited a considerably higher initial seam strength than those bonded with ABA. However, the seam strength of NABA-bonded specimens decreased more rapidly with increasing FTCs compared to ABA-bonded specimens. Despite this, after 200 FTCs, the seam strength of NABA-bonded specimens remained higher than that of ABA-bonded specimens. The FTCs led to a reduction in seam strength for specimens bonded with NABA with a maximum decrease of 58.5%, whereas they resulted in an increase in seam strength for specimens bonded with ABA with a maximum increase of 80.6%.

The seam strength of specimens bonded with NABA did not decrease but increased slightly due to FTCs. In contrast, the seam strength of ABA-bonded specimens increased initially when subjected to fewer than 150 FTCs but declined sharply after 200 FTCs. Although the seam strength of specimens bonded with NABA was still higher after 200 FTCs, from a long-term perspective, ABA-bonded specimens may exhibit a longer service life based on their change trend.

## 4. Discussion

The experimental results demonstrate that wetting and FTCs significantly affect the seam strength of specimens bonded with ABA and NABA. Combining Equations (1)–(3), the seam efficiency, actually the ratio of the maximum tension of seamed specimens to that of seamless specimens, does not accurately reflect its true value. In addition, the calculation of seam efficiency fails to consider the seam width, which experimental analysis has confirmed to be a critical factor affecting seam strength.

During the seam tensile test, the interface between the adhesive and GMB at the seams exhibits shear stress, the magnitude of which is influenced by both the specimen width and the seam width. In contrast, seamless specimens are subjected to axial tensile stress. Consequently, the ratio of the shear stress at the seams to the tensile stress in seamless specimens is utilized as an evaluation index for seam efficiency, defined as follows:

(4) $$\eta =\frac{100\times \tau_{max}}{\sigma_{yie}}$$

(5) $$\tau_{max}=\frac{P}{2Bb}$$

(6) $$\sigma_{yie}=\frac{T}{Bh}$$

where η is the seam efficiency, $\tau_{max}$ is the maximum shear stress at the seams, $\sigma_{yie}$ is the maximum tensile stress of seamless specimen, P  is the maximum tension of seamed specimens, T  is the yield load of seamless specimens, B  is the width of the specimen, b is the seam width, and h is the thickness of seamless specimens.

The maximum shear stress of seamed specimens and yield stress of seamless specimens were calculated by Equations (5) and (6), respectively, and the results are described in Table 5 and Table 6. The seam efficiency was determined according to Equation (4).

**Figure 8:**
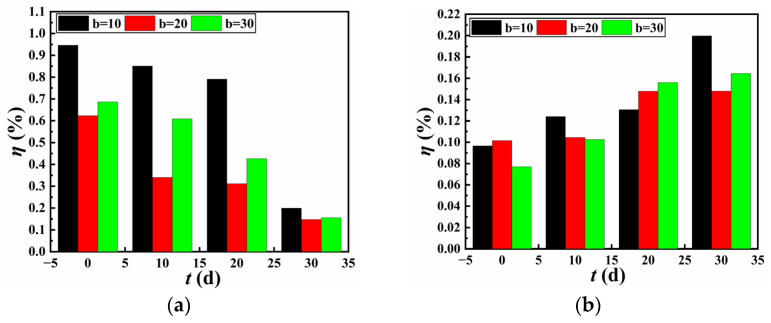
Relationship between seam efficiency and wetting time: (**a**) NABA; (**b**) ABA.

Figure 8 presents the relationship between seam efficiency at different seam widths and wetting time for specimens bonded with NABA and ABA, respectively. As the wetting time increased, the seam efficiency of specimens bonded with NABA decreased. The highest reduction in seam efficiency occurred at a seam width of 10 mm, dropping from 0.95% to 0.2%. The seam efficiency reached its maximum at a seam width of 10 mm and its minimum at 20 mm. After a wetting period of 30 d, no significant variation in seam efficiency was observed (the seam efficiency ranges from 0.2% to 0.15%), with the highest seam efficiency still occurring at a seam width of 10 mm. In contrast, the seam efficiency of specimens bonded with ABA increased with wetting time. Initially, there was little difference in seam efficiency among different seam widths (the seam efficiency was in the range of 0.10% to 0.08%). However, after 30 days of wetting, the seam efficiency at a seam width of 10 mm was the highest (0.2%), followed by 30 mm, and 20 mm was the lowest. Consequently, while the initial seam efficiency of NABA-bonded specimens was higher than that of ABA-bonded specimens (the maximum values were 0.95% and 0.10%), it gradually became lower than that of ABA-bonded specimens as the wetting time increased to 30 days.

**Figure 9:**
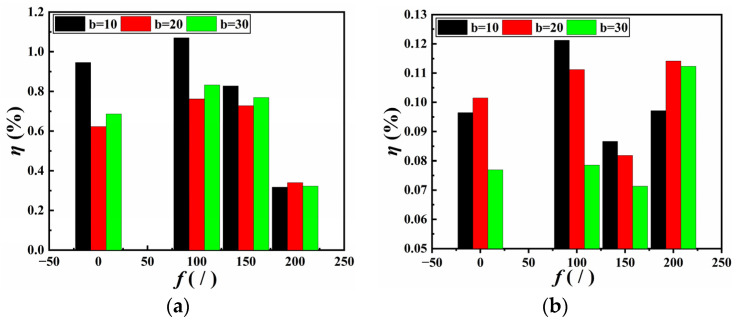
Relationship between seam efficiency and number of FTCs: (**a**) NABA; (**b**) ABA.

Figure 9 illustrates the relationship between seam efficiency at different seam widths and FTCs for specimens bonded with NABA and ABA, respectively. The maximum values of the initial seam efficiency for NABA-bonded specimens and ABA-bonded specimens were 0.95% and 0.10%, respectively. The As shown, an increase in FTCs resulted in an increase followed by a reduction in the seam efficiency of specimens bonded with NABA. For FTCs below 200, the highest seam efficiency was observed at a seam width of 10 mm, while the lowest was at 20 mm. No significant variation in seam efficiency was noted at 200 FTCs. When the seam width was 10 mm, the reduction in seam efficiency was the greatest, dropping from 1.07% to 0.32%. For specimens bonded with ABA, the seam efficiency initially increased with FTCs, then decreased, and subsequently increased again. However, the seam efficiency of specimens after subjected to 200 FTCs remained slightly higher than their initial values for three seam widths. Despite the gradual decrease in seam efficiency for NABA-bonded specimens, it remained higher than that of ABA-bonded specimens subjected to 200 FTCs. The highest seam efficiency after 200 FTCs for NABA-bonded specimens and ABA-bonded specimens were 0.34% and 0.11%, respectively.

The findings indicate that after wetting and FTCs, the seam efficiency of specimens bonded with NABA was minimally influenced by seam width. Whereas the seam efficiency of specimens bonded with ABA showed improvement, however, it remained below 1.2%, an extremely small value. The seam efficiency calculated using Equation (4) primarily depended on the interface shear stress between the adhesive and the GMB at the seams, with failure occurring at the seams when this shear stress reached its maximum. The degradation in seam efficiency induced by wetting and FTCs can be attributed to variations in the mechanical properties on the interface between the adhesive and the GMB.

**Figure 10:**
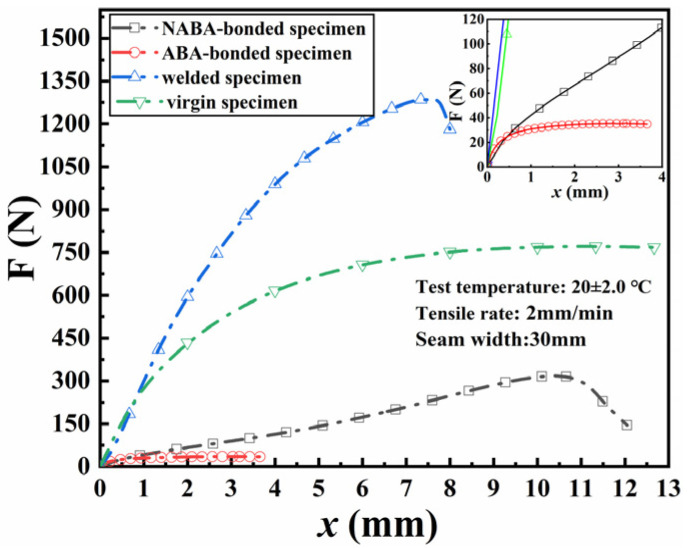
Axial tension-displacement curves for the bonded and welded specimens as well as the virgin specimen.

As evident in Table 5 and Table 6, the maximum shear stress of seamed specimens was significantly lower than the yield stress of seamless specimens. This failed to meet the specification requirement that the value should not be less than 80% of the tensile strength of seamless specimens. Figure 10 illustrates the axial tension versus displacement curves for the bonded and welded specimens (with a seam width of 30 mm) as well as the virgin specimen. The results showed that the yield deformation of the NABA-bonded specimens was significantly greater than that of the ABA-bonded, virgin, and welded specimens, indicating superior ductility, but the yield strength was relatively low. Although the break strength of the welded specimens exceeded that of the virgin specimen, the deformation at break was notably reduced. Both welding and bonding processes caused some degree of damage to the deformation of the virgin specimen; however, current standards prioritized strength retention over deformation. Therefore, welded seams were considered superior to bonded seams in terms of overall performance.

Consequently, bonding method in critical engineering locations was not recommended. For non-essential structural components where welding implementation was challenging but repair and replacement were relatively straightforward, the bonding method can be used with caution.

## 5. Conclusions

This paper presents an experimental investigation into the mechanical properties of HDPE GMB seams by bonding. The seam tensile tests were carried out under wetting and FTCs conditions. Based on the experimental data presented in this paper, the following conclusions are made:

(1) The results measured by the modified specimens can accurately represent the real seam strength, and the seam efficiency stipulated in the specification is updated.
(2) Under wetting conditions, the seam strength of specimens bonded with ABA increased as wetting time increased (with a maximum increase of 113.8%). Conversely, for specimens bonded with NABA, the seam strength decreased with increasing wetting time (with a maximum decrease of 93.4%). The seam strength of both specimens exhibited an increase with seam width. Prolonged wetting resulted in the seam strength of NABA-bonded specimens becoming lower than that of ABA-bonded specimens over time, indicating superior seam quality for ABA-bonded specimens under wetting conditions.
(3) The FTCs led to a reduction in seam strength for specimens bonded with NABA with a maximum decrease of 58.5%, whereas they resulted in an increase in seam strength for specimens bonded with ABA with a maximum increase of 80.6%. After 200 FTCs, the seam strength of NABA-bonded specimens remained higher than that of ABA-bonded specimens.
(4) The specimens bonded with NABA exhibited a reduction in seam efficiency due to wetting and FTCs, whereas those bonded with ABA showed a slight improvement. However, the improved seam efficiency remained below 1.2%, an extremely small value. Consequently, the mechanical properties of ABA-bonded specimens were superior to those of NABA-bonded specimens.
(5) The seam width had minimal effect on seam efficiency under wetting and FTCs conditions. Therefore, it can be reasonably selected based on engineering practice, provided that seam quality is ensured.
(6) Given that the axial tensile strength of bonded specimens was significantly lower than that of seamless specimens, bonding method in critical engineering locations was not recommended. However, the bonding method can be used with caution for non-essential structural components where welding implementation was challenging but repair and replacement were relatively straightforward.

The findings contribute to a deeper understanding of the tensile mechanical properties of HDPE GMBs at the seams by bonding method, and provide valuable guidance for GMBs as anti-seepage materials in engineering design and construction. However, this study does not investigate the hydraulic characteristics and aging resistance of GMBs at the bonded seams, which may result in changes in the seam strength, subsequently affecting the durability of GMBs. Therefore, further research should be conducted as follows:

(1) To examine whether the impermeability of bonded seams satisfies the requirements for safe engineering operation, and to identify the influencing factors and underlying mechanisms.
(2) To identify the physical and mechanical properties as well as the hydraulic characteristics of specialized adhesives used for GMBs.
(3) To investigate the aging resistance of GMBs at seams and predict their service life using a numerical model.

## Figures and Tables

**Table 1:** Characteristic parameters of GMB and special adhesives.

Name	Mass per Unit Area	Thickness	Tensile Strength at Yield	Tensile Strength at Break	Tensile Strain at Yield
	g/m^2^	mm	N/mm	N/mm	%
HDPE GMB	2400.00	0.5	7.320	20.00	146.99
ABA	9296.66	1.0	4.796	—	—
NABA	4187.77	0.8	5.294	—	—

**Table 2:** Tensile rates recommended in existing test methods.

Methods	SL 235-2012 [32]	GB/T 16989-2013 [34]	ASTM D7408-12 [35]	ASTM D4885-01 [30]	ASTM D638-22 [36]	ASTM D6693 [37]
Tensile rate (mm/min)	20	20	50.8	10.0	5.0	50

**Table 3:** Seam strength of bonded specimens after wetting.

Seam Width (mm)	Wetting Time ti/d				△t1 (t1–t0)	△t2 (t2–t0)	△t3 (t3–t0)	△t4 (t2–t1)	△t5 (t3–t2)	Unit
	t0 = 0	t1 = 10	t2 = 20	t3 = 30						
10 (ABA)	0.0993	0.1275	0.1343	0.1855	0.0282	0.035	0.0862	0.0068	0.0512	kN/m
20 (ABA)	0.2088	0.2148	0.3039	0.4041	0.0060	0.0951	0.1953	0.0891	0.1002	kN/m
30 (ABA)	0.2374	0.3164	0.4814	0.5076	0.0790	0.244	0.2702	0.1650	0.0262	kN/m
10 (NABA)	0.9733	0.8753	0.8132	0.0615	−0.098	−0.1601	−0.9118	−0.0621	−0.7517	kN/m
20 (NABA)	1.2825	0.7033	0.6416	0.1952	−0.5792	−0.6409	−1.0873	−0.0617	−0.4464	kN/m
30 (NABA)	2.1185	1.8780	1.3153	0.1943	−0.2405	−0.8032	−1.9242	−0.5627	−1.121	kN/m

**Table 4:** Seam strength of bonded specimens subjected to FTCs.

Seam Width (mm)	FTCs Ni/Number				△N1 (N1–N0)	△N2 (N2–N0)	△N3 (N3–N0)	△N4 (N2–N1)	△N5 (N3–N2)	Unit
	N0 = 0	N1 = 100	N2 = 150	N3 = 200						
10 (ABA)	0.0993	0.1569	0.1206	0.1236	0.0576	0.0214	0.0244	−0.0362	0.003	kN/m
20 (ABA)	0.2088	0.2287	0.2117	0.2905	0.0199	0.0029	0.0817	−0.017	0.0788	kN/m
30 (ABA)	0.2374	0.328	0.277	0.4287	0.0906	0.0396	0.1913	−0.051	0.1517	kN/m
10 (NABA)	0.9733	1.4896	1.0715	0.4044	0.5163	0.0982	−0.5689	−0.4181	−0.6671	kN/m
20 (NABA)	1.2825	2.1205	1.8842	0.8655	0.8380	0.6017	−0.4170	−0.2363	−1.0187	kN/m
30 (NABA)	2.1185	3.4754	2.9862	1.2324	1.3570	0.8677	−0.8861	−0.4892	−1.7538	kN/m

**Table 5:** Maximum shear stress/yield stress under different wetting times.

Seam Width (mm)	Wetting Time (d)				Unit
	0	10	20	30	
10 (ABA)	0.0099	0.0128	0.0134	0.0205	MPa
20 (ABA)	0.0104	0.0107	0.0152	0.0152	MPa
30 (ABA)	0.0079	0.0105	0.0169	0.016	MPa
10 (NABA)	0.0973	0.0813	0.0875	0.0061	MPa
20 (NABA)	0.0641	0.0321	0.035	0.0098	MPa
30 (NABA)	0.0706	0.0438	0.0626	0.0065	MPa
0 (SS)	10.2839	10.2123	10.2678	10.2864	MPa

Note: All data in the table, except for the yield stress of the seamless specimen, represent shear stress values. SS represents the seamless specimen.

**Table 6:** Maximum shear stress/yield stress under different FTCs.

Seam Width (mm)	FTCs/Number			Unit
	100	150	200	
10 (ABA)	0.0121	0.0157	0.0124	MPa
20 (ABA)	0.0114	0.0106	0.0145	MPa
30 (ABA)	0.0109	0.0092	0.0143	MPa
10 (NABA)	0.149	0.1072	0.0404	MPa
20 (NABA)	0.106	0.0942	0.0433	MPa
30 (NABA)	0.1158	0.0995	0.0411	MPa
0 (SS)	13.914	12.9374	12.7203	MPa

Note: All data in the table, except for the yield stress of the seamless specimen, represent shear stress values. SS represents the seamless specimen.

## Data Availability

The original contributions presented in this study are included in the article. Further inquiries can be directed to the corresponding author.
